# Neural Activities in Multiple Rat Brain Regions in Lithium-Pilocarpine-Induced Status Epilepticus Model

**DOI:** 10.3389/fnmol.2019.00323

**Published:** 2020-01-15

**Authors:** Jingjing Fan, Wei Shan, Huajun Yang, Fei Zhu, Xiao Liu, Qun Wang

**Affiliations:** ^1^Department of Neurology, Beijing Tiantan Hospital, Capital Medical University, Beijing, China; ^2^National Center for Clinical Medicine of Neurological Diseases, Beijing, China; ^3^Beijing Institute for Brain Disorders, Beijing, China

**Keywords:** seizure model, brain activity, neural activation, status epilepticus, mossy fiber sprouting

## Abstract

To clarify the different regional brain electroencephalogram (EEG) activities and biochemical responses in seizure and epilepsy models, we assessed the EEG and c-Fos immunolabeling characteristics in a lithium-pilocarpine-induced status epilepticus (SE) model and pentylenetetrazol (PTZ)-induced seizure model. The regional brain activities were evaluated by EEG and c-Fos immunolabeling. ZnT3 immunostaining was performed to observe hippocampal mossy fiber sprouting (MFS) within 7 days after the induction of SE in the lithium-pilocarpine model. The EEG recordings showed distinctive features of activation in different brain areas. With the aggravation of the behavioral manifestations of the seizures, the frequency and amplitude of the discharges on EEG gradually increased. SE was eventually induced and sustained. The labeling of c-Fos was enhanced in the cortex and hippocampal CA1, CA3, and dentate gyrus (DG); however, compared to the PTZ-induced seizure model, c-Fos staining could only be observed in the striatum and thalamus in the lithium-pilocarpine-induced epilepsy model. In each brain region, prominent c-Fos labeling was observed 2 h and 4 h after the induction of SE or seizures and diminished at 24 h. During the lithium-pilocarpine-induced chronic epilepsy phase after SE induction, MFS was observed 7 days after SE and was accompanied by the dynamic evolution of epileptic EEG activities. These findings validated the lithium-pilocarpine-induced SE model as an epilepsy model with a specific spatial-temporal profile of neural activation. The EEG characteristics and c-Fos expression patterns differ from those presented in a previous study using a PTZ-induced seizure model. Hippocampal mossy fiber spouting might be associated with spontaneous seizures during the chronic phase and can be detected at least within 1 week by ZnT3 staining after stimulation.

## Introduction

Epilepsy is a complex neurological disease with a variety of manifestations, etiologies and pathogenesis. Although neuronal overexcitation is considered an essential feature across different types of epileptic seizure models at the cellular level, each type or model has a specific neuronal electroencephalogram (EEG) or c-Fos expression pattern due to different mechanisms in each model.

Temporal lobe epilepsy (TLE) is the most common type of partial epilepsy (Téllez-Zenteno and Hernández-Ronquillo, 2012), which is characterized by recurrent spontaneous convulsive seizures that are associated with significant neuron loss and morphological changes affecting mainly mesial temporal structures such as the amygdala and hippocampal formation (French et al., 1993; Engel, 1996; Bernasconi et al., 2003). Status epilepticus (SE) is one of the most severe conditions of epilepsy, which is caused by a hypersynchronous activation of neurons. If SE is untreated, it could result in severe brain damage or death (Hellier and Dudek, 1999; Trinka et al., 2012; Leitinger et al., 2019; Upadhya et al., 2019). In the lithium–pilocarpine model, pilocarpine, which is a direct-acting cholinergic muscarinic agonist, is administered after pretreatment with lithium to induce continuous seizures; this model is a popular model of acute SE and chronic TLE, which develops within days to weeks after experimental SE (Lévesque et al., 2016). The epilepsy model induced by pilocarpine could reproduce the main clinical and pathophysiological characteristics of human TLE, i.e., hippocampal sclerosis, mossy fiber sprouting (MFS), cell dispersion in the dentate gyrus (DG), and gliosis (Leite et al., 1990; Curia et al., 2008). Additionally, this model could reproduce the severe neuronal damage in the mesial structures of the brain (Kandratavicius et al., 2014).

In spite of numerous available antiepileptic drugs (AEDs), about 20–30% of the patients with TLE suffer refractory epileptic seizures (Goldenberg, 2010). The Lithium-Pilocarpine model shares features of human TLE and SE and, thus, serves as a valuable tool for the development of drugs and therapeutic management of refractory seizures and SE (Brandt et al., 2015). Growing evidence based on studies involving animal models indicates that multiple mechanisms are involved in epileptogenesis. The cellular changes include neurodegeneration, neurogenesis, axonal sprouting, dendritic remodeling, invasion of inflammatory cells, gliosis, angiogenesis, and alterations in the extracellular matrix (Pitkänen and Lukasiuk, 2009). Seizure activity could also develop as a result of alterations in the efficacy of excitatory or inhibitory synapses between neurons (Beck and Yaari, 2008). The prevention of epileptogenesis is supposed to be a key approach to TLE treatment. Therefore, it is essential to understand the detailed pathogenic mechanisms of the process.

However, few studies have explored the electrophysiological profiles of the multiple brain regions involved and neural activation pattern using both EEG and biochemical methods in the Lithium-Pilo model, and no studies have indicated the different EEG characteristics and c-Fos expression pattern among different seizure or epilepsy models. Hence, in the present study, we established a Lithium-Pilocarpine-induced SE model in rats, mapped different regional brain activities through a combination of EEG responses and c-Fos expression in different brain regions and compared these activities with a pentylenetetrazol (PTZ)-induced seizure model. We aimed to systemically investigate the spatial-temporal profile of c-Fos expression and its concordance with behavioral and electrophysiological features in different brain regions during the whole process of SE induction to improve our understanding and rational application of this model.

## Materials and Methods

This study was not pre-registered.

### Animals

Male Sprague–Dawley (SD) rats (RGD Cat# 10395233, weighing 250–280 g) were provided by Beijing Vital River Laboratory Animal Technology Co., Ltd. and were in-housed under controlled conditions (22 ± 1°C, 12 h/12 h light/dark cycle, lights on at 7:00 a.m.) with *ad libitum* access to water and food. The ethics research committee of Capital Medical University approved all experiments. The experiments were performed following the principles outlined in the Animal Research: Reporting of *In vivo* Experiments (ARRIVE) guidelines, the Basel declaration^1^ and the National Institutes of Health Guide for the Care and Use of Laboratory Animals (NIH Publications No. 8023, revised 1978). The Replacement, Refinement, and Reduction of Animals in Research (3R) concept was considered when planning the experiments to minimize the animal numbers and pain or discomfort of the animals. When neurochemical experiments were carried out, rats were decapitated under deep anesthesia with 10% Chloral hydrate to reduce animal pain. Totally 62 rats used in this study, more detail information can be found in the tables and figures.

All the experiments were performed between 9 o’clock am to 17 o’clock pm.

### Time-Line Diagram

For the acute seizure behavior observation, the behavioral changes induced by pilocarpine or PTZ during the process of SE induction or seizure response were observed within 90 min. For the continuous behavioral observations (colonic stage) after SE were performed in the morning from 10 o’clock to 12 o’clock during the following 1–3 weeks.

The rat behavior analysis was performed within 2 h after the saline, pilocarpine or PTZ treatment in the colonic stage. EEG signals were recorded for 14 days after implanting. For the c-Fos mapping experiments, it has been performed at 2 h, 4 h, 24 h or 7 days respectively after drug treatment. More detail information refers to the result parts and Figure legendary parts.

### Electrode Implantation and EEG Recording

Electrode implantation and EEG recordings were implemented according to the procedures described previously (Yang et al., 2019). The rats were anesthetized by inhalation of isoflurane. Compound needle electrodes consisting of Tungsten wires were implanted and fixed to the skull with dental acrylic. The free end of the wire was soldered to a multipin connector and secured by dental acrylic. A 16-electrode referential montage was implanted at the unilateral cortex, hippocampal CA1, CA3, and DG, amygdala, and striatum and used for the EEG recording. All regions mentioned above were located based on the Paxinos and Watson rat brain atlas (Paxinos and Franklin, 1997). The anteroposterior, mediolateral, and dorsoventral coordinates relative to bregma were as follows (in mm): cortex: 1.80, 1.6, and 0.8; nucleus accumbens (NAc): 2.0, 1.5, and 6.3; striatum: 1.4, 2.5, and 4.8; Amg: −1.20, 4.0, and 8.2; DG: −3.84, 1.7, and 3.8; CA1: −3.84, 2.0, and 2.4; and CA3: −3.84, 2.6, and 3.0.

### Seizures and SE Induction

The rats were injected i.p. with 127 mg/kg lithium chloride. After approximately 18 h, the rats received methylscopolamine (1 mg/kg s.c., Sigma-Aldrich, St. Louis, MO, USA) to limit the undesirable peripheral effects of pilocarpine. SE was induced after approximately 30 min by the administration of pilocarpine (30 mg/kg i.p., Abcam). The control group received lithium chloride and saline instead of pilocarpine. PTZ (Sigma, St. Louis, MO, USA) was dissolved in 1X phosphate buffer saline (PBS). The rats were treated with 55 mg/kg PTZ (i.p.) for the seizure induction. The intensity of the seizures was classified according to Racine’s scale as follows: stage 1, immobility, eyes closed, and facial clonus; stage 2, head nodding and more severe facial clonus; stage 3, clonus of one forelimb; stage 4, rearing with bilateral forelimb clonus; and stage 5, generalized tonic-clonic seizures. If SE lasted over 1.5 h, 10% chloral hydrate was administered i.p. to terminate SE to minimize mortality due to long-lasting seizures.

### C-Fos Immunostaining

C-Fos immunostaining was performed to map c-Fos expression in different regions of the brain according to a procedure described in previous studies (Szyndler et al., 2009). Histology: In total, 15 animals were divided into five groups (three per group) to determine c-Fos expression in the control group and 2 h, 4 h, 24 h, and 7 days after SE onset. The rats were deeply anesthetized with 10% chloral hydrate and transcardially perfused with PBS, followed by 4% paraformaldehyde (PFA). The brains were extracted, fixed in 4% PFA overnight, and then stored in 20% and 30% sucrose at 4°C until cutting. Coronal sections were sliced at a 30-μm thickness with a freezing microtome (Leica Biosystems, CM3050, Nussloch GmbH, German). Then the sections were washed using 0.1 M PB and rinsed twice for 10 min. After that, the sections were incubated with 0.3% hydrogen peroxide for 30 min. After washing with 0.1 M PB twice, the sections were preincubated in PB containing 0.1% bovine serum albumin and 0.5% Triton-X (PBST)for 1 h to block the nonspecific binding sites. Then the sections were washed with PB twice and incubated with primary anti-c-Fos antibody (Santa Cruz, RRID:AB_2106765, 1:3,000 dilutions in blocking buffer) at 4°C for 24 h. After the primary incubation, the sections were then washed with PB twice and incubated with biotinylated secondary antibody (Vector Laboratories, RRID:AB_2313606, goat anti-rabbit IgG, diluted 1:200 in PBST) for 90 min at room temperature. Afterward, the sections were rinsed with PB twice and reacted with the ABC solution (avidin-biotin complex; RRID:AB_2336819, Vectastain, Vector Laboratories, diluted 1:800 in PBST) for 120 min. Subsequently, the sections were incubated for 3 min in a 3,3P-diaminobenzidine (DAB) solution (0.05% DAB, 0.001% H_2_O_2_ with metal intensification using cobalt chloride and nickel ammonium sulfate). Finally, the sections were rinsed with PB three times, mounted on slides, dehydrated and coverslipped. The regions of interest (ROI) include the cortex, hippocampal subfields (DG, CA1, and CA3) and the striatum. All ROI were delineated based on the Paxinos and Watson rat brain atlas (Paxinos and Franklin, 1997).

### ZnT3 Immunostaining

At different times after the SE induction, the animals were processed for ZnT3 immunostaining to visualize MFS as previously described (Ishihara and Fukuda, 2016). In total, 12 animals were divided into four groups (three per group) to observe MFS on days 1, 3 and 7 and a saline control group. Serial sections of 30 μm thickness were sliced in a horizontal plane using a freezing microtome from a brain block containing the entire hippocampal formation. Following cryo-protection in 20% sucrose for 24 h and 30% sucrose for 24 h in PBS, the sections were placed on aluminum foil, rapidly frozen in liquid N2 vapor, rapidly thawed in O.C.T. compound (Sakura, Japan), and subsequently processed for immunohistochemistry as previously described (Ishihara and Fukuda, 2016). A rabbit anti-ZnT3 primary antibody (Synaptic Systems, RRID:AB_2274984, dilution 1:500 in PBST) and biotinylated secondary antibody (Vector Laboratories, RRID:AB_2313606, goat anti-rabbit IgG, diluted 1:200 in PBST) were used. The immunostained slices were scanned with a 10× objective under a microscope (Olympus BX 61) equipped with a high-sensitive CCD camera. Most images were obtained using the Metamorph image acquisition software (Molecular Devices, Sunnyvale, CA, USA), and analyzed with Adobe Photoshop (CS4). The images of slices were overlaid with the corresponding atlas maps (Paxinos and Franklin, 1997), enabling us to outline the different brain regions.

### Statistical Analysis

The digitally stored EEG records were scanned to detect and measure the seizure episodes using NeuroExplorer 5.0 (Nex Technologies). In this study, a seizure episode was defined as either generalized or focal epileptiform activity occurring with an amplitude unambiguously greater than the background. The latent period of a certain seizure stage was defined as the time from the pilocarpine injection to the first episode of that seizure stage. The mean latent periods, durations and amplitudes were calculated using SPSS 19.0 and are reported as the mean ± SD.

## Results

For the acute seizure behavior observation, the behavioral changes induced by pilocarpine or PTZ during the process of SE induction or seizure response were observed. At 10–60 min after the pilocarpine or PTZ injection, the animals began to show piloerection, akinesia, progressive body tremor, salivation, and minor seizures (Stage 1–2) that evolved to repeated generalized clonic seizures (forelimb clonus with rearing and falling, Stage 3–4) or finally SE (Stage 4–5) 30–60 min after injection.

**Figure 1:**
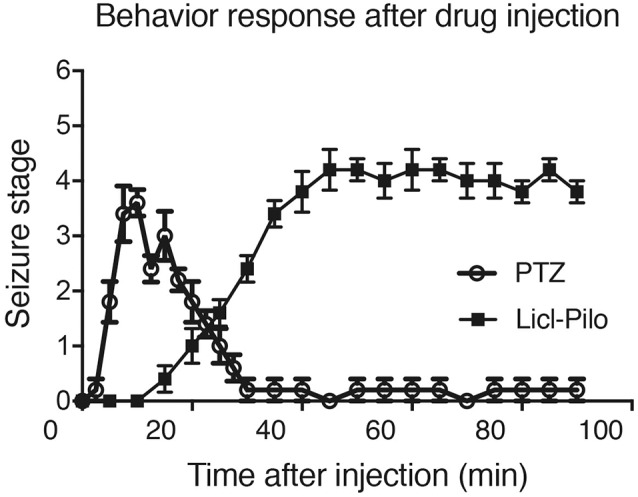
Rat behavior responses after pentylenetetrazol (PTZ) induced seizure response or Licl-pilocarpine epilepsy response. The seizure behavior responses started within 10 min after the injection of PTZ and reach to the peak within 20 min. The last seizure response is about 10 min. However, the model injected with pilocarpine, mainly ranged from Stage 1–2 and occasionally reached Stage 3 or 4. SE was induced 30–60 min after the administration of pilocarpine (*n* = 5 rats for each group, totally 10 rats were used in this experiment).

For the continuous behavioral observations (colonic stage) after SE were performed in the morning from 10 o’clock to 12 o’clock during the following 1–3 weeks. After a silent period of 5–7 days (during which no clear behavioral seizures were observed), a state of chronic epilepsy characterized by spontaneous recurrent seizures (mostly Stage 1–3) was observed (Figure 1). Most seizures apparently were not related to environmental stimulation.

### EEG Changes in Different Brain Regions in Response to SE Induction

**Figure 2:**
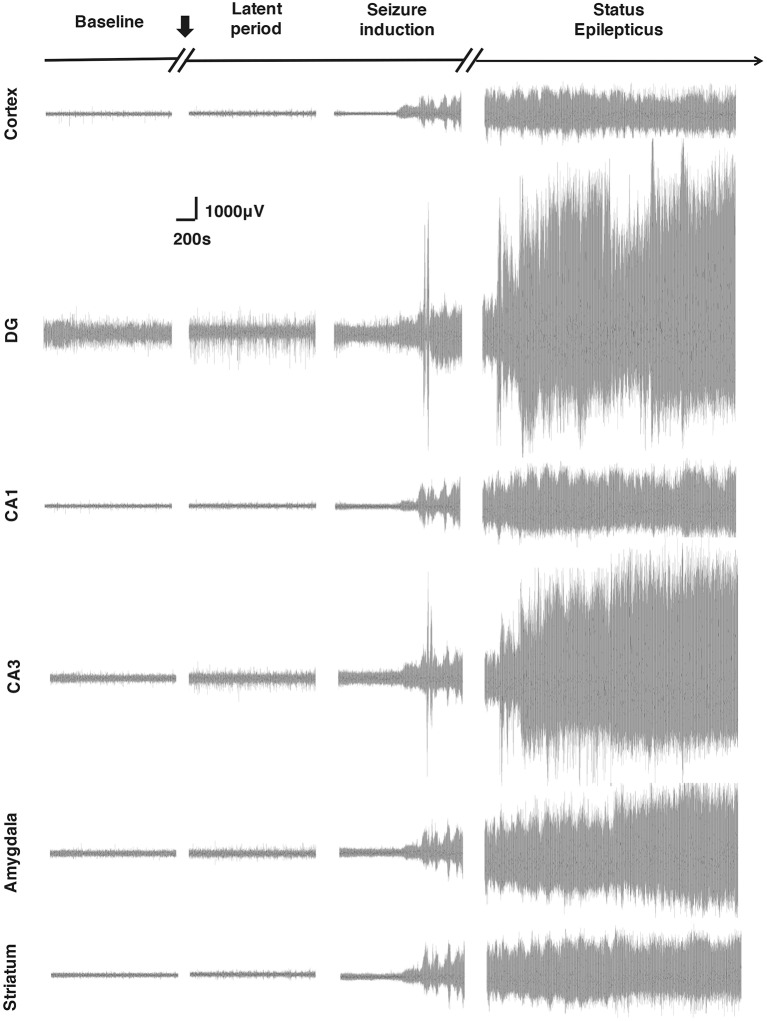
Electroencephalogram (EEG) changes in different brain regions in response to status epilepticus (SE) induction. The most robust EEG response to the seizures was observed in the dentate gyrus (DG). The EEG response in the CA3 was also significant. A relatively moderate response was observed in the cortex, CA1, amygdala and striatum (*n* = 3 rats for each group, totally three rats were used in this experiment).

In this experiment, through EEG monitoring, we found that different brain regions showed different degrees of activation in response to SE induction, indicating that the Cortex, amygdala, hippocampal formation, and striatum participated in the process of Lithium-Pilocarpine-induced SE in different degrees (Figure 2, Table 1). The hippocampal DG exhibited the most robust activation, and CA3 exhibited a significant EEG response to the seizure activity, while the activation of hippocampal CA1 the, Cortex, the amygdala and the striatum was relatively moderate on EEG. Notably, during the induction period, the EEG response in hippocampal DG and CA3 was relatively moderate when Stage 1–2 seizures occurred; once Stage 3–4 seizures and SE were reached, the response in hippocampal DG and CA3 became extremely strong on EEG. These findings suggested that compared with the PTZ model, different brain regions exhibit specific electrophysiological profiles in the Lithium-Pilocarpine model, which might reflect their respective roles in the occurrence and development of seizures and SE. It has been reported that the hippocampal area is more likely involved in high-stage seizures, such as clonic or tonic-clonic seizures (Szyndler et al., 2009; Samokhina and Samokhin, 2018). The hippocampus has a relatively low epileptogenic threshold, which might explain the robust EEG response in CA3 and DG compared with that observed in the other brain areas. The degree of EEG activity in different brain regions might be associated with their involvement in seizures of certain types or severities and their excitability in response to epileptogenic stimuli (Yang et al., 2019).

**Table 1:** Electroencephalogram (EEG) characteristics of different stages of seizures in the cortex (*n* = 3 for this experiment).

Racine’s stage	Latent period (s)	A_min_ (μV)	A_max_ (μV)	A_mean_ (μV)	Waveform features
Baseline	N/A	27	84	54.8 ± 9.5	4–5 Hz
Stage 1–2	1,218 ± 395.3	54	256	153.0 ± 35.1	3–4 Hz rhythmic S
Stage 3	1,734 ± 501.8	97	615	341.8 ± 45.2	3–4 Hz rhythmic PS
Stage 4	1,958 ± 550.7	114	1,150	676.9 ± 106.1	clusters of continuous S (6–8 Hz)
Stage 4 SE	2,261 ± 643.3	243	1,697	997.5 ± 178.2	continuous S (8–10 Hz)
Stage 5 SE	2,786 ± 755.5	281	2,211	1,295.6 ± 223.4	continuous S (8–10 Hz)

*A_min_, minimum amplitude; A_max_, maximum amplitude; A_mean_, mean amplitude; S, spikes; PS, poly-spikes*.

### Behavioral and EEG Changes Induced by Different Stages of Seizures

**Figure 3:**
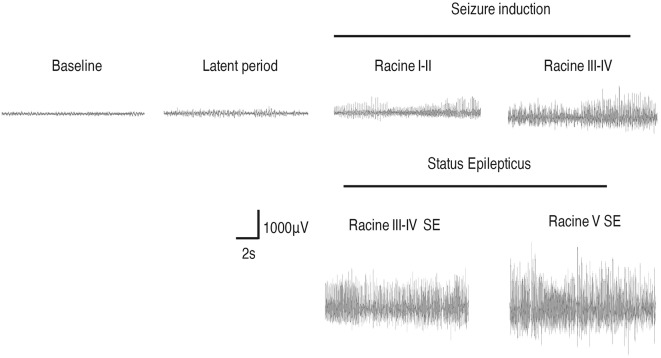
EEG changes during the process of Lithium-Pilocarpine-induced SE (cortex). During the seizure induction period, Stage 1–2 seizures were usually characterized by low-amplitude rhythmic spikes (3–4 Hz) on EEG. Stage 3–4 seizures were characterized by rhythmic poly-spikes (3–4 Hz) or small clusters of continuous spikes (6–8 Hz) and usually occurred in a rhythmic manner. Once SE was induced, long-lasting continuous spikes were observed with a higher amplitude and frequency (8–10 Hz; *n* = 3 rats for each group, totally three rats were used in this experiment).

We monitored the behaviors of the animals and assigned scores to the seizure levels. The seizures started 15–30 min after the injection of pilocarpine and mainly ranged from Stage 1–2 and occasionally reached Stage 3 or 4. SE was induced 30–60 min after the administration of pilocarpine and was characterized by long-lasting persistent Stage 3–5 seizures. EEG monitoring of the brain ROI described above (see “Materials and Methods” section) was conducted simultaneously with the behavioral monitoring. The EEG showed various responses in a stage-dependent manner (Figure 3, Table 2). Synchronous and relatively consistent general features were observed across different brain areas. During the seizure induction period, Stage 1–2 seizures were usually characterized by low-amplitude rhythmic spikes (3–4 Hz) on EEG. Stage 3–4 seizures were characterized by rhythmic poly-spikes (3–4 Hz) or small clusters of continuous spikes (6–8 Hz) which usually occurred in a rhythmic manner. Once SE was induced, long-lasting continuous spikes were observed with a higher amplitude and frequency (8–10 Hz). These findings suggested that a certain dynamic evolutionary process occurred in the behavioral and EEG features in this model.

**Table 2:** The amplitude of EEG waveforms during different stages of seizures in multiple brain regions (*n* = 3 for this experiment).

Racine’s stage	A_mean_ in different brain regions (μV)					
	Cortex	CA1	CA3	DG	Amygdala	Striatum
Baseline	54.8 ± 9.5	46.3 ± 7.4	126.7 ± 43.4	298.9 ± 68.8	76.5 ± 19.6	54.8 ± 9.5
Stage 1–2	153.0 ± 35.1	171.5 ± 41.5	442.5 ± 102.1	701.9 ± 189.2	317.8 ± 77.8	325.0 ± 80.1
Stage 3	341.8 ± 45.2	419.4 ± 82.2	1,251.3 ± 348.1	1,994.3 ± 578.2	482.4 ± 106.2	486.8 ± 112.2
Stage 4	676.9 ± 106.1	769.9 ± 158.4	2,293.0 ± 681.2	3,734.6 ± 882.4	914.0 ± 160.9	881.9 ± 142.1
Stage 4 SE	997.5 ± 178.2	1,254.5 ± 252.2	3,243.9 ± 932.2	5,108.5 ± 1,112.3	1,193.2 ± 234.4	1,014.5 ± 218.2
Stage 5 SE	1,295.6 ± 223.4	1,704.9 ± 396.3	4,053.4 ± 1,270.3	6,482.5 ± 1792.9	1,766.5 ± 403.1	1,797.6 ± 423.4

*A_mean_, mean amplitude*.

### Mapping c-Fos Expression in Different Brain Regions

**Figure 4:**
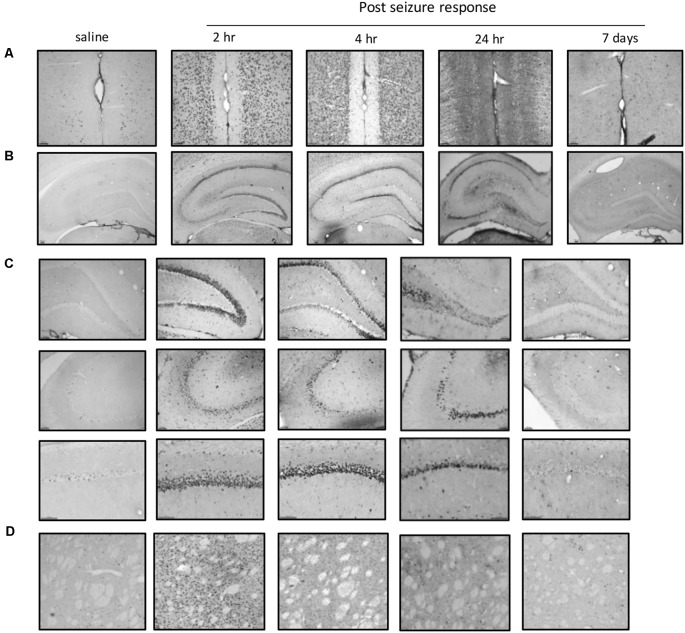
Mapping of c-Fos positive neurons in different brain areas at different time points after SE induction. **(A)** Significant c-Fos immunoreactivity could be observed at 2 h and 4 h. The immunoreactivity appeared to diminish at 24 h and completely returned to the basal level on day 7. The main structures recruited in the Lithium-Pilocarpine-induced SE model included the Cortex, hippocampal formation (CA1, CA3 and DG) and striatum. **(A)** Cortex; **(B)** hippocampus; **(C)** hippocampal subfields; **(D)** striatum (*n* = 3 rats for each group, totally 15 rats were used in this experiment).

To further investigate the regional brain activities in the cellular biochemical response, we used c-Fos to identify the anatomical structures in which the neurons were activated. C-Fos immunostaining was performed 2 h, 4 h, 24 h, and 7 days after SE induction and compared to the saline control group (Figure 4). Significant c-Fos immunoreactivity could be observed at 2 h and 4 h. This immunoreactivity appeared to diminish at 24 h and completely returned to the basal level on day 7. A systemic mapping of c-Fos expression in different brain regions was performed using immunostaining. The results showed that the main structures recruited in the Lithium-Pilocarpine-induced SE model included the Cortex, the hippocampal formation (CA1, CA3 and DG) and the striatum, which exhibited significant c-Fos immunolabeling.

### Regional Brain Activity in the Striatum Based on EEG Recording and c-Fos Expression

**Figure 5:**
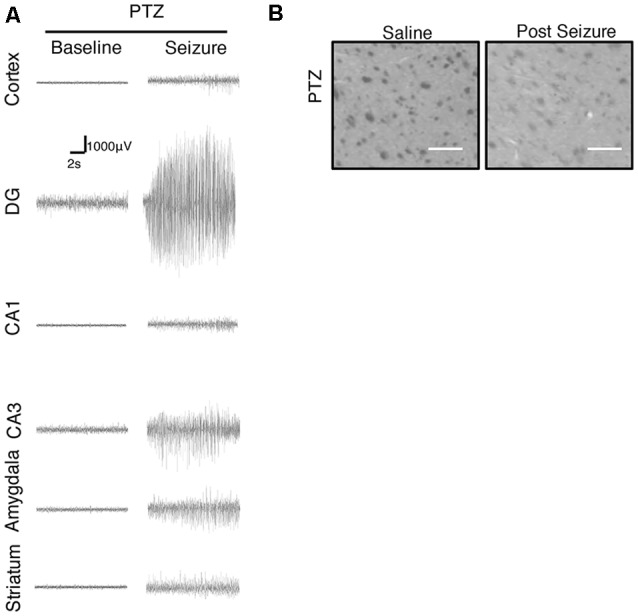
Regional brain activities in the PTZ seizure model by EEG and c-Fos expression. **(A)** EEG activities of baseline and Stage 4–5 seizures in different brain regions. **(B)** c-Fos expression in the striatum (2 h vs. control), no significant c-Fos immunoreactivity was observed in the PTZ model (*n* = 3/4 rats for each group, totally seven rats were used in this experiment).

**Figure 6:**
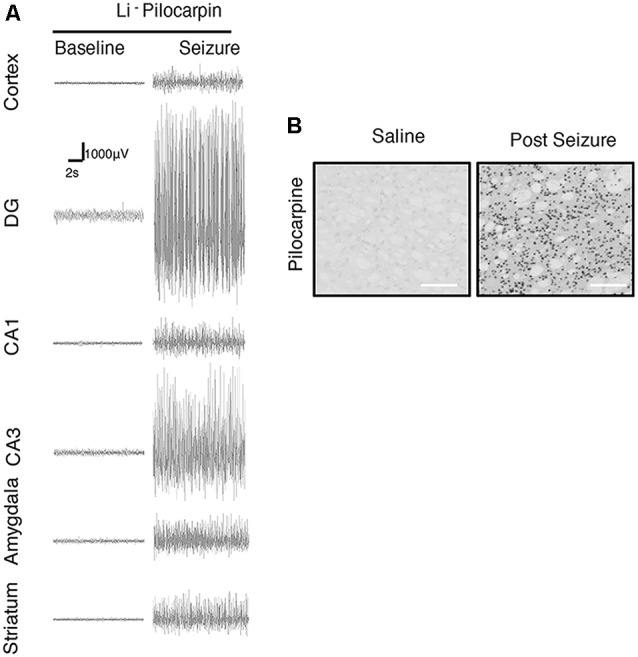
Regional brain activities in the Lithium-Pilocarpine models by EEG and c-Fos expression. **(A)** EEG activities of baseline and Stage 4–5 seizures. **(B)** c-Fos expression in the striatum (2 h vs. control); positive c-Fos immunoreactivity was observed in the Lithium-Pilocarpine model (*n* = 3/4 rats for each group, totally seven rats were used in this experiment).

We further focused on the regional brain activities in the striatum, which was investigated through EEG recording and c-Fos expression. We found positive c-Fos immunoreactivity and EEG responses in the striatum (Figures 2, 5, 6, Tables s 3, 4), suggesting that the striatum was activated during the process of SE induction and persistence. The striatum has been shown to exert an inhibitory effect in seizures involving the limbic system, especially those with motor symptoms. This structure is considered to be activated as negative feedback to seizure activities. The result differs from our previous data from a PTZ-induced acute seizure model in which no significant c-Fos immunoreactivity was observed in the striatum (Figures 5, 6).

**Table 3:** Comparison of EEG waveform amplitude in multiple brain regions in pentylenetetrazol (PTZ) models (*n* = 3 for each group).

	Baseline Pilocarpine	Stage 4–5 seizure Pilocarpine
Cortex	54.8 ± 9.5	1,295.6 ± 223.4
DG	298.9 ± 68.8	6,482.5 ± 1,792.9
CA1	46.3 ± 7.4	1,704.9 ± 396.3
CA3	126.7 ± 43.4	4,053.4 ± 1,270.3
Amygdala	71.5 ± 12.6	1,766.5 ± 403.1
Striatum	54.8 ± 9.5	1,797.6 ± 423.4

**Table 4:** Comparison of EEG waveform amplitude in multiple brain regions in Lithium-Pilocarpine models (*n* = 3 for each group).

	Baseline PTZ	Stage 4–5 seizure PTZ
Cortex	52.7 ± 8.1	547.4 ± 112.9
DG	295.4 ± 54.6	3,741.5 ± 902.5
CA1	44.8 ± 7.6	517.4 ± 102.1
CA3	123.1 ± 47.2	1,723.1 ± 435.5
Amygdala	71.0 ± 11.4	870.2 ± 145.2
Striatum	53.4 ± 11.3	617.1 ± 131.9

### Dynamic Evolution of EEG Characteristics and Mossy Fiber Sprouting in the Lithium-Pilocarpine Model of Chronic Epilepsy

**Figure 7:**
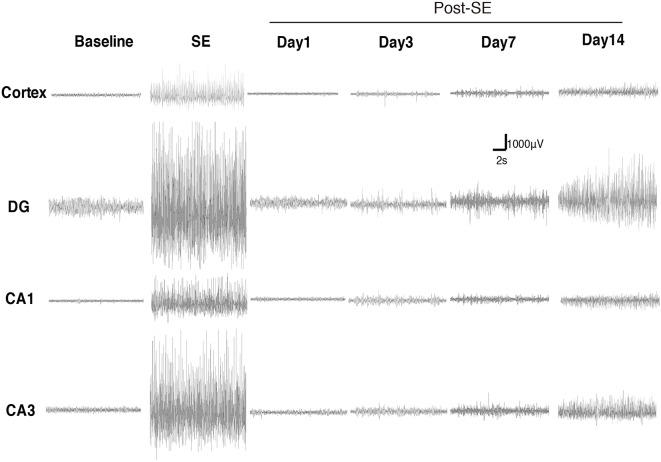
Dynamic changes in EEG activities in the Cortex and hippocampal subfields during the chronic phase after Lithium-Pilocarpine SE induction. On day 1 after SE induction, the EEG showed no significant change compared with baseline. On day 3 after SE, the seizures were symptomatic; scattered low-amplitude spikes were observed on EEG in the Cortex, and similar EEG changes were observed synchronously in the hippocampal subfields during the seizures. On day 7, Stage 1–2 seizures occurred; spikes were observed more frequently in the Cortex and hippocampal subfields, with higher amplitudes. On day 14, Stage 3 seizures occurred; rhythmic or continuous spikes (6–8 Hz) with a higher amplitude were observed (*n* = 3 rats for each group, totally 12 rats were used in this experiment).

**Figure 8:**
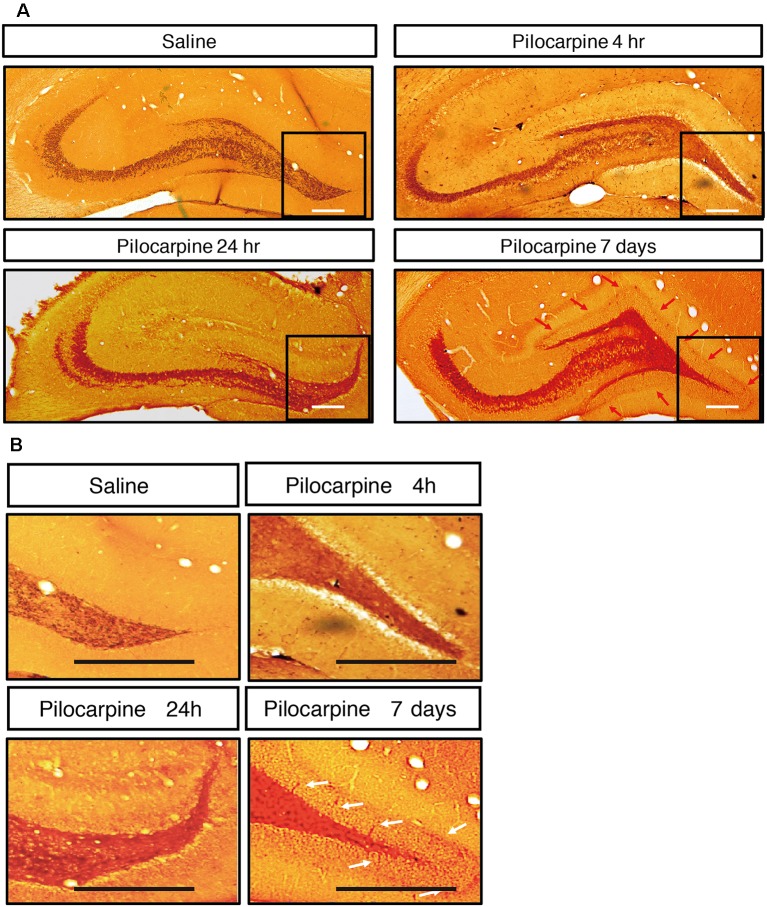
**(A)** Mossy fiber sprouting in hippocampus after treatment. **(B)** Zoom-inimage in DG region. ZnT3 staining of mossy fibers in the hippocampal area at different time points after SE induction. Positive staining of mossy fibers in the lamella adjacent to the DG granular cell layer could be observed on the 7th day after SE, while such changes were not observed 4 h and 24 h after SE (*n* = 3 rats for each group, totally 12 rats were used in this experiment).

We monitored the dynamic changes in EEG activities in the Cortex and hippocampal subfields during the chronic phase after SE induction (Figure 7). On the 1st day after SE induction, the EEG showed no significant change compared with baseline. The animals exhibited relatively silent behaviors, but no seizure episodes were observed. On day 3 after SE induction, scattered low-amplitude spikes were observed on EEG in the Cortex, and similar EEG changes were observed synchronously in the hippocampal subfields. No definite convulsive symptoms were observed behaviorally simultaneously with the spikes. On day 7 after SE, the spikes were observed more frequently in the Cortex and hippocampal subfields, and their amplitudes were higher than those on the 3rd day. An episodic behavioral arrest or head nodding (Stage 1–2 seizures) were observed simultaneously with the spikes on EEG. On day 14, episodic limb clonus (Stage 3 seizures) could be observed occasionally, and rhythmic or continuous spikes (6–8 Hz) with a higher amplitude were observed on EEG simultaneously with limb clonus. Compared with the control group, we also observed MFS in the hippocampal DG area using ZnT3 staining 4 h, 24 h and 7 days after SE induction (Figure 8). Positive staining of mossy fibers in the lamella adjacent to the DG granular cell layer could be observed on the 7th day after SE, which is suggestive of the early phase of MFS, while such changes were not observed 4 h and 24 h after SE.

## Discussion

In the present study, the behavioral and electroencephalographic expression of Lithium-Pilocarpine-induced seizures and SE suggested that the higher stage seizures, the higher frequency, and amplitude of EEG. The duration is much longer and could not be in spontaneous elimination within 30 min. In our experiment, based on the EEG response and c-Fos expression, the brain structures recruited in Lithium-Pilocarpine-induced SE included the Cortex, the amygdala, hippocampal DG, CA1, and CA3 and the striatum. This finding reflected the neural activation pattern in high-stage generalized seizures and SE and the neural circuit involved in the generation and propagation of seizures.

The association between the clinical manifestations and EEG expression was relatively typical and somewhat similar to the EEG changes recorded in epileptic patients (Asadollahi et al., 2018). We observed a specific temporal and spatial profile in Lithium-Pilocarpine-induced SE based on the clinical manifestations and EEG changes. The different intensities of the epileptic EEG activities in different brain regions might also reflect different excitabilities during seizure development. The response in hippocampal DG and CA3 became extremely strong on EEG on Stage 3–4 seizures and SE.

Our previous data obtained from a PTZ-induced acute seizure model suggested that regional brain activation occurs in the piriform cortex, prefrontal cortex, amygdala, and hippocampus, which is similar to the Lithium-Pilocarpine model. However, the activation of the striatum significantly differed between these two models. The EEG response was relatively limited, and c-Fos expression was not observed in the PTZ model. Previous studies investigating seizures induced by different doses of PTZ in rats showed that seizures of different severities have different influences on the pattern of c-Fos expression (André et al., 1998). In adult rats, absence-like seizures induced by a relatively low-dose of PTZ exhibited low to moderate immunoreactivity of c-Fos in brain areas including the piriform cortex, the cingulate cortex, the amygdala, the hypothalamus and thalamic nuclei, but showed no expression of c-Fos in any hippocampal substructures. Clonic seizures induced by a moderate-dose of PTZ led to stronger c-Fos expression than absence-like seizures in all cortical and forebrain areas and showed moderate to significant immunolabeling of c-Fos in most hippocampal substructures. SE induced by high-dose PTZ showed moderate to significant c-Fos expression in all brain areas, including subcortical structures, such as the striatum (André et al., 1998; Szyndler et al., 2009).

However, in both models, there are some discrepancies in the activation degrees of the brain structures between the EEG activity and c-Fos immunoreactivity. A possible explanation for this discrepancy is that EEG signaling not only depends on the local signals in certain brain regions but also affected by the signal input from the connection from adjacent or remote areas or passing nerve fibers. This finding indicated the limitation of EEG in reflecting regional brain activities in spite of high sensitivity and good temporal resolution. On the other hand, c-Fos expression, as a hysteretic biochemical response of neuronal activation, is not sensitive enough to reflect relatively weak activation. Thus, neuro-electrophysiological monitoring and quantitative investigations of biochemical indicators need to be combined to accurately reflect regional brain activation patterns mediated by seizure activities.

The hippocampal formation contributes to the development of seizures. Based on the important role of the hippocampus in epilepsy. We observed the spontaneous recurrent seizures and MFS. In this study, we observed c-Fos expression in a group of brain structures during the acute phase following seizures in the Lithium-Pilocarpine model, while during the chronic phase, the EEG started to show epileptiform activities from day 3 after SE. On days 7–14, the epileptiform activities became more frequent and prominent. Meanwhile, the animals began to show spontaneous seizure episodes (Stage 1–3). In the MFS observations, abnormal stained mossy fibers were observed in the adjacent layers of the granular cells in the hippocampal DG on the 7th day after SE, which was considered an early change in MFS. This phenomenon suggested that the spontaneous recurrent seizures during the chronic phase might be associated with hippocampal MFS. However, the observation period of the chronic phase in this experiment was relatively short and not long enough for significant typical pathological changes to develop in the cortex or hippocampal structures. Therefore, whether MFS participates in the recurrence of spontaneous epileptic seizures as the pathological basis of chronic epilepsy still needs a longer behavioral observation period. The EEG and pathological changes during the chronic phase after SE should be explored in future studies.

## Conclusion

To the best of our knowledge, this study is the first to map regional brain activities in Lithium-Pilocarpine-induced seizures and SE in rats in a detailed and systematic way. This study established a model of SE that was induced after Lithium-Pilocarpine administration. The EEG response and c-Fos expression showed that distinctive features of activation could be observed in different brain areas. A certain group of structures, including the Cortex, hippocampal CA1, CA3 and DG, and the striatum, was recruited during the course of this type of chemically induced seizures. This information indicates the existence of anatomical seizure circuits that interact to facilitate and modulate seizure activities in the brain. The distinctive characteristics of neural activation observed on EEG and in the c-Fos immunolabeling showed the different roles of brain structures in neural circuits involved in seizure generation, propagation and modulation. We also found the development of epileptiform EEG activities, which was accompanied by the early stage of MFS, during the chronic phase after SE, which might provide some evidence of MFS as a pathological basis of spontaneous chronic seizures. Furthermore, the quantification of regional brain activation in different seizure models is necessary for a thorough understanding of the features and underlying mechanisms of different types of seizures.

## Data Availability Statement

The datasets generated for this study are available on request to the corresponding author.

## Ethics Statement

The animal study was reviewed and approved by the ethics research committee of Capital Medical University.

## Author Contributions

JF and WS contributed equally to this work and should be considered first co-authors, and they participated in literature search, figures, study design, data collection, data analysis, data interpretation, writing, critical approval of the final report, and funding. QW had full access to the data and take responsibility for the integrity of the data and the accuracy of analysis. XL, FZ, and HY participated in data collection, writing and critical approval of final report.

## Conflict of Interest

The authors declare that the research was conducted in the absence of any commercial or financial relationships that could be construed as a potential conflict of interest.

## Footnotes

^1^http://www.basel-declaration.org

## Abbreviations

AEDs: anti-epileptic drugs
AF: Alexa Fluor
CA1: Cornu Ammonis area 1
CA3: Cornu Ammonis area 3
DAB: 3,3P-diaminobenzidine
DG: dentate gyrus
EEG: electroencephalogram
GABA: γ-aminobutyric acid
IEG: immediate early gene
NAc: nucleus accumbens
PB: phosphate buffer
PBS: phosphate-buffered saline
PBST: phosphate-buffered saline with Tween
PFA: paraformaldehyde
PTZ: pentylenetetrazol
ROI: regions of interest
SE: status epilepticus
TLE: temporal lobe epilepsy.
